# Runge psychoses

**DOI:** 10.1007/s00737-016-0678-5

**Published:** 2016-10-08

**Authors:** Ian Brockington

**Affiliations:** University of Birmingham, Edgbaston, B15 2TT, Birmingham,, UK

**Keywords:** Runge psychosis, Hysterical psychosis, Verbigeration, Prepartum psychosis, Menstrual psychosis, 1st trimester of pregnancy

## Abstract

This article describes periodic monthly psychoses that develop during the early months of pregnancy. It is probable that these are a variety of menstrual psychosis.

## Cases in the literature

A hundred years ago, the Kiel nosologist Runge (1911), who wrote one of the most comprehensive accounts of *Generationpsychosen*, described this patient with five brief episodes of a polymorphic psychosis in the first half of pregnancy:

A 20-year-old unmarried woman, whose mother and sister were mentally ill, became pregnant, with her last period at the beginning of March. Four weeks later, as soon as she missed her next period, she became disturbed with restlessness, pressure of speech and destructiveness; this lasted 8 days. She relapsed a month later: she tore her clothes and ran naked into the street, cycled off in garters and slip, hit out, bit, scratched and smashed windows, sang and spoke incoherently. On May 7th she was admitted to hospital: she was disorientated, heard voices and said she had seen the Devil. By the 11th (after 4 days) she had recovered. On June 6th she relapsed, recovering on the 17th. On July 5th she relapsed, recovering by the 12th. On August 13th she relapsed, recovering on the 21st. She gave birth on November 30th (case 11 on pages 640–641 and 679–681).

Provided that this was a full-term pregnancy, her first episode was in the first month of gestation. Runge made a diagnosis of hysterical psychosis, but Ewald (1928) wrote,

Runge gave an instructive example of a hysterical-delirious confusional state, which affected a single woman, starting in the first month and returning every 4 weeks, at a time her menses were expected.

He was the first to claim that this is a variant of menstrual psychoses.

Twenty years later, a second, French, case was described, but with only two episodes (Dupouy. 1930):

In January, a 19-year old single woman became pregnant for the first time. Towards the end of February she developed confusion and agitation, for which she was admitted to hospital: she showed continuous verbigeration and a state of dreamy confusion, lasting 15 days, after which she became lucid and calm. About 4 weeks later she had a new attack of excitement with agitation, euphoria, logorrhoea, incoherence, punning and joking, disrobing, shouting, singing, food refusal and insomnia. After 8 days it cleared up again, and she returned to normal, remaining well for the rest of the pregnancy (case 4).

Twelve other authors have described manic or cycloid episodes, starting in the first month of pregnancy, which may have been single Runge episodes (Moll 1822, Dubrisay 1858, Ribell 1877, Ripping 1877, Boullé 1899, Boutet 1911, Hoch and Kirby 1919, Fumarola 1935, Visscher 1949, Moisan 1982. One author had two cases (Schorer 1972). One (Bourson 1958) had two possible Runge episodes in different pregnancies.

## Personal experience

In my series of 321 childbearing psychoses, there are six mothers with possible Runge episodes. This is one, a patient who also had two postpartum psychoses with menstrual relapses:

A mother, who had five pregnancies and 18 miscarriages, suffered psychiatric complications with each pregnancy. The first birth was followed by 9 months depression (without psychotic features). The second was followed by a delusional depression, with onset on day 12. During her third pregnancy, she became manic at 6 months gestation, with a recurrence starting 3 days after the birth; she improved, then relapsed with every menstruation, at intervals of exactly 28 days. Her fourth birth was followed, 3 days later, by a psychosis lasting only 4 days. She gave birth for the fifth time at 25 weeks gestation and again developed a psychosis on the third day, followed by brief menstrual relapses. None of her 18 miscarriages had psychiatric complications, but, during one of these brief pregnancies, at 6 weeks gestation, she had a 4-day psychosis, in which she heard frightening voices, and believed the baby was conceived by the Devil; 2 weeks later she miscarried.

Another became “extremely high, on cloud 9 and talking loads” and was bleeding heavily when 5–6 weeks pregnant. A third mother was hospitalized with mania and then had a 2-month termination of pregnancy. The fourth became manic soon after conception and had three relapses during that pregnancy. The fifth had a more continuous illness: the first manic episode developed 6 weeks after conception, and she continued to suffer brief episodes from then on, during two pregnancies and two postpartum periods. This is the sixth patient:

A 16-year old suffered from two acute psychotic episodes with cycloid features, 1 month apart. While at a Christian retreat, she believed a woman had put a curse on her. The world had come to an end, and Jesus had come to take away the believers, but left her behind. Her fear was so terrible that her father (whom she believed to be an angel or a ghost) had to stay up with her all night; she was about to give birth to a dog. When her mother washed her hair, she was terrified that she was trying to drown her; and a family picnic was a pretext for sacrificing her. Colours were too bright, noises and voices distorted, and there were odd smells around. Seen by a psychiatrist, she was in a semi-stupor, preoccupied and perplexed. After a 9-day illness she recovered but relapsed a month later. She remembered these episodes as a time of fear and confusion. Both episodes began 2–3 days before her menses.

At the age of 24, during her 1st pregnancy, she menstruated once. She was delivered by emergency Caesarean section, and breast-fed for 6 weeks. On day 6 she suddenly developed mania: she thought she was in Hell, had been carrying triplets and had an abortion forced on her. Her role was to convert the rest of the world to Christianity before the 2nd coming. Her thoughts were racing and she had pressure of speech, talking nonsense with neologisms and echolalia—‘speaking in tongues’. Her diary was full of bizarre pseudoscientific jottings. She was overfamiliar, distractible and disinhibited. She recovered quickly, but relapsed six times—44, 82, 115, 137, 163 and 198 days after the birth. There was statistical confirmation that these were premenstrual episodes.

The Runge psychosis developed during her 2nd pregnancy. She and her husband were in New Zealand when she conceived about December 21st. At the end of the year she flew home, a 36-h flight. She was already high on arrival. Admitted to hospital on January 3rd, she was excited and confused, overactive, sleepless and elated. Her mind was being directed by a massive computer, and her actions affected everyone. She was preoccupied with the periodic table; all atoms had to have a fixed pattern otherwise the ceiling beams would collapse. The pregnancy was confirmed and treatment withheld, but within 3 days she improved. On 28th she relapsed, higher than ever; her condition deteriorated so rapidly that she was sedated with haloperidol; by February 10th she had recovered. On 22nd she relapsed again, recovering by March 8th. She had two minor relapses on March 24th and April 26th, after which she remained well during the rest of her pregnancy. In August she gave birth to her 2nd child. Two days later she developed puerperal mania, with two relapses.

During an observation period of 41 years after the first episode, she had other brief episodes. It is not certain that any of her episodes were unrelated to the reproductive process.

This mother suffered two episodes of early onset postpartum mania, both with a relapsing pattern. There was statistical evidence of menstrual relapses following the first pregnancy, and she had two adolescent premenstrual episodes. During her second pregnancy, she had four or five manic episodes during the early months.

## Discussion

Menstrual psychosis is defined as the occurrence of acute, usually brief, periodic bipolar or cycloid episodes occurring in rhythm with the menstrual cycle. Since women, between the menarche and the menopause, spend at least one third of their lives in the premenstrual or menstrual phase, a considerable number of episodes must occur, with dating of episode and the onset of menstrual bleeding, before one can be reasonably sure that the association is not coincidental. Four or five episodes, supplemented by collateral evidence, are insufficient for proof, but the association can be upheld ‘on the balance of probability’. Runge’s case, and one in my experience, reach this level, and justify offering ‘the Runge psychoses’ as a clinical hypothesis.

There is much evidence that menstrual psychosis is associated with abnormal menstruation, including luteal cell defects and anovulatory cycles; episodes also occur during amenorrhoea (Brockington 2008, Brockington 2016). The Runge psychosis is another example of a periodic psychosis developing during anomalous ‘menstrual’ activity. Menstruation is suppressed during pregnancy, probably by high levels of chorionic gonadotropins, as well as oestrogen and progesterone. But menstruation-like bleeding undoubtedly occurs. A Münster Inaugural-Dissertation (Lehsau 1948) collected 45 cases. A German review of six papers, with a total of 60,000 births, estimated the frequency as 1 % of pregnancies: 11/64 cases had six or more bleeds, nine of which were no less copious than normal menstruation (Goecke 1951). Outside the German literature, it is seldom mentioned, but an American paper reported that 8/221 expectant mothers reported bleeding at the time the first post-conception menses was due; one bled for five consecutive days (Harville et al. 2003), and a Japanese author reported a mother who bled regularly every month during two pregnancies (Sato and Yamane 1971). It is not known why menstruation-like bleeding occurs during pregnancy.

With, at the most, 15 examples in the literature, this is not a common phenomenon. But it is important to bring it to the attention of obstetricians and psychiatrists, because it may be less rare than it appears. Two mothers in my series had other evidence of a menstrual psychosis, and this supports the hypothesis that Runge psychoses are menstrual phenomena. It would be strengthened by the observation of cases in which the onset of psychosis and menstrual bleeding was simultaneous. This has not yet been reported.
